# Modulation of early non-rapid eye movement slow wave activity by ketamine in treatment-resistant depression

**DOI:** 10.1038/s41386-026-02465-4

**Published:** 2026-06-16

**Authors:** Nadia Hejazi, Mina Kheirkhah, Brady Riedner, Qiaoping Yuan, Robin Chholak, Reza Momenan, Gregory Jones, David Goldman, Carlos A. Zarate

**Affiliations:** 1 Experimental Therapeutics and Pathophysiology Branch, Intramural Research Program, National Institute of Mental Health, National Institutes of Health, Bethesda, MD USA; 2 Department of Psychiatry and Psychotherapy, Jena University Hospital, Jena, Germany; 3 Department of Psychology, University of Marburg, Marburg, Germany; 4 Exercise Psychology Laboratory, Department of Kinesiology, University of Wisconsin-Madison, Madison, WI USA; 5 National Institute on Alcohol Abuse and Alcoholism, National Institutes of Health, Bethesda, MD USA

**Keywords:** Depression, Electrophysiology

## Abstract

Slow-wave activity (SWA), a key indicator of sleep homeostasis, is often diminished in individuals with treatment-resistant depression (TRD). Ketamine, a rapid-acting antidepressant, increases SWA during the first period of non-rapid eye movement (NREM1) sleep. However, research into how ketamine affects sleep and its connection to treatment outcomes in TRD has been limited by small sample sizes and a lack of comparison with healthy volunteers (HVs). This placebo-controlled study compared the effects of ketamine on NREM1 SWA in a large sample of unmedicated TRD patients (n = 91; 51 F/40 M) and HVs (n = 42; 23 F/19 M). Linear mixed-effects models and regression analyses, with post-hoc testing (paired *t*tests and Wilcoxon matched-pairs signed rank tests), were used to assess condition-related effects across baseline, ketamine, and placebo in TRD and HV participants. Age-related moderation of ketamine-associated NREM1 SWA changes and condition-related changes in sleep variables were also examined. At baseline, TRD patients had lower NREM1 SWA than HVs. Ketamine, but not placebo, increased NREM1 SWA in TRD patients, particularly in responders. In contrast, ketamine had no effect on NREM1 SWA in HVs. Following ketamine, TRD patients also showed significant increases in total sleep time and sleep efficiency and a reduction in sleep latency. In TRD patients, the ketamine-related increase in NREM1 SWA diminished with increasing age. Together, the results indicate that ketamine’s antidepressant effects appear closely associated with its ability to modulate early SWA. These effects may also be linked to ketamine’s ability to improve sleep architecture in TRD. Clinical Trials Identifier: www.clinicaltrials.gov, NCT00088699, NCT01204918.

## Introduction

Electroencephalographic (EEG) slow-wave activity (SWA), also referred to as delta power (0.5–4 Hz), occurs during non-rapid eye movement (NREM) sleep and is homeostatically regulated [1]. According to the two-process model, sleep is regulated by the interaction between two complementary processes: Process S, the homeostatic sleep drive, and Process C, the circadian rhythm [2]. Process S reflects sleep pressure accumulation during wakefulness that dissipates during sleep and is indexed by SWA. In contrast, Process C is driven by the body’s internal biological clock and modulates the timing of sleep and wakefulness across the approximately 24-h cycle.

During NREM sleep, cortical neurons alternate between synchronized up states and silent down states, a dynamic reflected in EEGs as SWA [3, 4]. SWA is highest early in sleep, particularly following prolonged wakefulness, and serves as a sensitive measure of sleep homeostasis. According to the synaptic homeostasis hypothesis, wakefulness is associated with net synaptic potentiation, whereas sleep promotes synaptic downscaling, accompanied by a progressive decline in SWA across the night [5, 6]. This daily cycle of synaptic strengthening and renormalization supports learning and plasticity, while sleep deprivation leads to saturated synaptic potentiation and impaired long-term potentiation (LTP)-like plasticity [7].

Major depressive disorder (MDD) is marked by impairments in both sleep quality and sleep homeostasis [8, 9], including consistently reduced SWA relative to healthy volunteers (HVs) [10–12]. These SWA deficits are equal or more pronounced in treatment-resistant depression (TRD) [13–15]. One proposed mechanism for this finding is deficient Process S, whereby the homeostatic sleep drive fails to adequately accumulate during wakefulness, resulting in attenuated SWA in early NREM sleep. Because SWA reflects synaptic plasticity and neuronal synchrony [14], its reduction may signal impaired synaptic function central to MDD pathophysiology [16].

The N-methyl-D-aspartate (NMDA) receptor antagonist ketamine exerts rapid antidepressant effects and has been shown to increase SWA in MDD patients [14, 17, 18]. This SWA enhancement has been linked to increased brain-derived neurotrophic factor (BDNF), implicating cortical plasticity and restoration of sleep homeostasis as part of ketamine’s therapeutic actions [14]. In addition, because aging is associated with reduced NREM SWA and diminished sleep-dependent synaptic plasticity, age-related declines in plasticity may moderate ketamine’s antidepressant and SWA-enhancing effects [13, 19].

To date, few studies have specifically examined SWA in TRD patients [13, 15, 20]. Furthermore, only one study to date has investigated the effects of ketamine on SWA during the first NREM sleep (NREM1) in TRD [14], and that study lacked a placebo control, did not include a comparison to HVs, and was limited in size (n = 30). Addressing these issues could clarify how ketamine modulates SWA in TRD and offer critical insights into ketamine’s underlying antidepressant mechanisms.

This study sought to investigate the impact of ketamine on SWA in TRD and HV participants. The working hypotheses were that: (1) at baseline, TRD patients would exhibit reduced SWA during NREM1 relative to HVs, reflecting an impaired buildup of homeostatic sleep pressure during wakefulness or a reduced capacity for neural synchronization, and (2) ketamine would increase NREM1 SWA in TRD patients compared to both baseline and placebo conditions; this increase would be most evident in TRD patients who responded to ketamine with improved depression rating scale scores. As a secondary aim, the study explored whether NREM1 SWA would remain unchanged in HVs post-ketamine, reflecting that ketamine-induced changes in NREM1 SWA do not generalize to healthy individuals. The study also explored whether age moderated ketamine-related changes in NREM1 SWA, with primary emphasis on TRD patients and secondary evaluation in HVs. As exploratory aims, the study also investigated whether ketamine’s effects on NREM1 SWA and other polysomnographic (PSG) measures differed between TRD patients and HVs; sleep-related variables of interest included: total sleep time (TST), sleep efficiency (SE), wake after sleep onset (WASO), slow wave sleep (SWS), and sleep latency (SL). Age moderation of ketamine-related SWA changes was evaluated separately in TRD responders and non-responders.

## Materials and methods

### Participants and study design

This study was a secondary analysis of 91 unmedicated adults diagnosed with treatment-resistant MDD (n = 64) or bipolar disorder (n = 27) (n = 91, 51 F/40 M, age 20–70, mean ± SD = 43.09 ± 12.61 years) and 42 HVs (23 F/19 M, age 20–58, mean ± SD = 34.14 ± 10.75 years) who were enrolled in two inpatient, randomized, controlled clinical trials conducted at the NIH Clinical Center in Bethesda, MD, USA between 2006 and 2018 (NCT00088699, NCT01204918). The two racemic ketamine (hereafter referred to as ketamine) trials were conducted under NIH protocol 04-M-0222. Findings from the primary analyses of these trials have previously been published [21, 22]. Full demographic and clinical characteristics are provided in Table S1; the CONSORT diagram appears as Fig. S1.

Briefly, TRD patients had a diagnosis of MDD or bipolar disorder without psychotic features, confirmed using the Structured Clinical Interview for DSM-IV Axis I Disorders (SCID–Patient Version) [23], a baseline Montgomery-Åsberg Depression Rating Scale (MADRS) [24] score≥20 or a 21-item Hamilton Depression Rating Scale [25] score>18, and a current depressive episode lasting at least four weeks. Diagnostic subtype was not included as a factor in the primary analyses. All patients had a documented history of nonresponse to two or more adequate antidepressant trials, as defined by the parent protocols; electroconvulsive therapy (ECT) counted as one treatment trial. Exclusion criteria included a DSM-IV diagnosis of substance abuse or dependence within the preceding three months, unstable medical illness, or obstructive sleep apnea (OSA). Screening for OSA included the STOP-BANG questionnaire [26], the Stanford Sleepiness Scale [27], and a review of external sleep apnea records when available. Comorbid anxiety disorders were not exclusionary.

All TRD patients were medication-free for at least two weeks prior to study participation (five weeks for fluoxetine, three weeks for aripiprazole) and were closely monitored as inpatients during the medication washout period. Participants were strictly prohibited from using sedatives (including benzodiazepines and non-benzodiazepine agents), melatonin, antihistamines, and stimulants; coffee consumption was permitted. The mean MADRS score for the TRD group was 33.27 ± 4.49, significantly higher than the MADRS score of HVs at baseline (1.22 ± 1.38) (Table S1). Mean body mass index (BMI) for TRD patients was 29.63 ± 6.85 (range:19.16–55.43). Antidepressant response to ketamine was defined as a ≥ 50% reduction in MADRS score 24 h post-ketamine. Responders (n = 37) and non-responders (n = 53) were classified accordingly (one TRD patient had missing response data).

HVs were aged 20–58, with BMI ranging from 19.39–34.70 (mean = 27.20 ± 4.52), had no current or past DSM-IV Axis I disorder as assessed via the SCID-Non-Patient version [23], and had no first-degree relatives with Axis I diagnoses. Of the 42 HVs enrolled, 22 received ketamine. All procedures were approved by the NIH IRB, and all participants provided written informed consent at the time of entry into their original study.

### Polysomnography (PSG)

In the studies from which these data were drawn, all participants completed at least four nights of laboratory-based PSG performed by sleep technicians. Night 1 served as an adaptation night to familiarize participants with the sleep lab environment and EEG setup, and Night 2 captured baseline sleep. Night 3 assessed the acute effects of either ketamine (0.5 mg/kg IV over 40 min) or placebo, administered in a randomized, double-blind manner, and Night 4 was the crossover condition two weeks later. The initial baseline night (Night 2) was used as the reference for both the ketamine and placebo conditions to minimize potential bias.

**Fig. 1:**
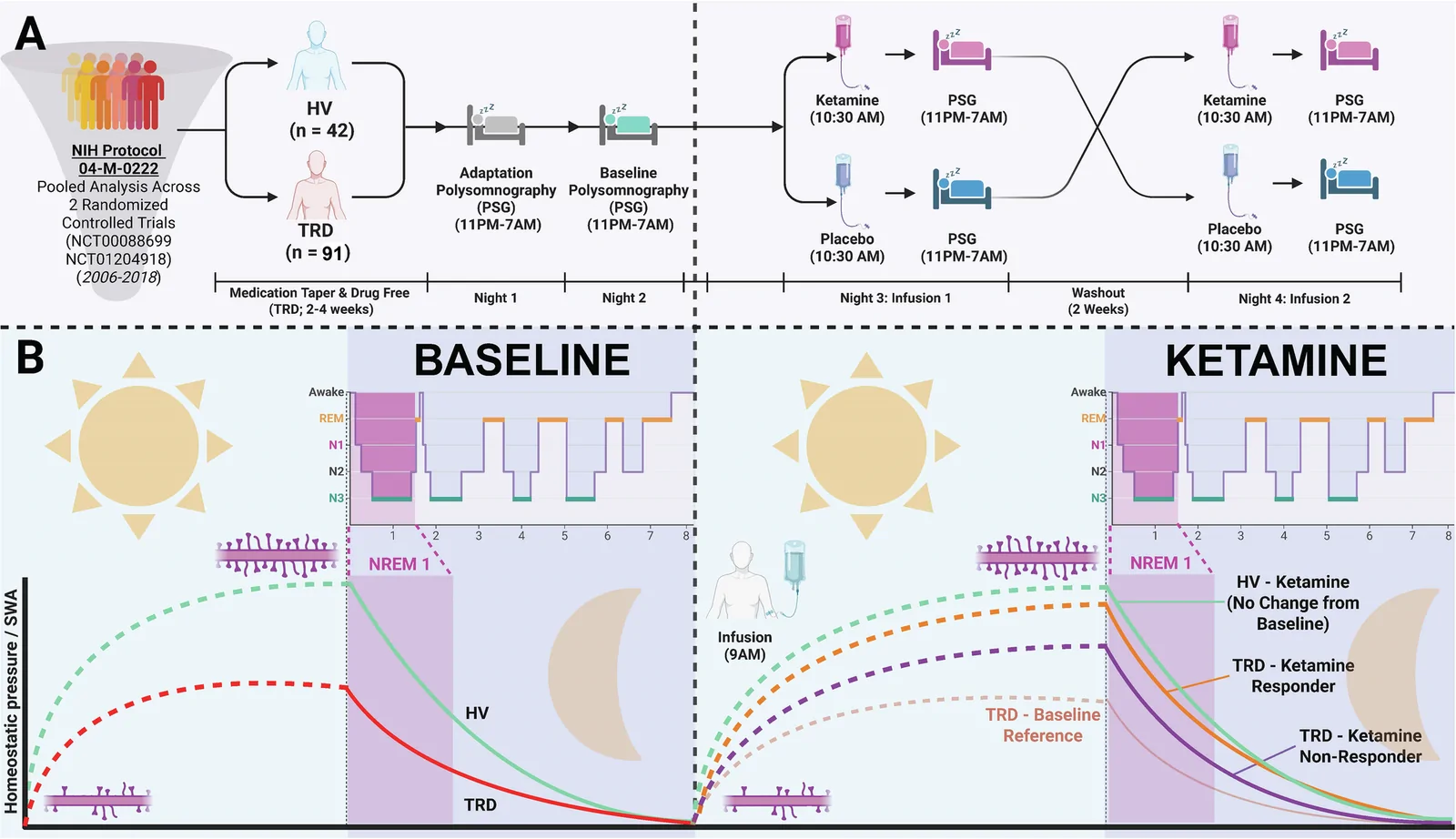
Clinical trial design for PSG data collection and treatment nights. **A** Clinical trial design for PSG data collection. The study data come from a pooled analysis of data collected in two randomized, placebo-controlled, crossover trials involving TRD patients and HVs. The protocol included medication tapering followed by baseline and post-infusion PSG recordings to assess sleep architecture after administration of intravenous ketamine or placebo. **B** Schematic of study rationale, hypothesis, and analysis. The curved colored lines represent hypothetical time courses of SWA and homeostatic sleep pressure, both of which increase throughout the course of a day (dashed lines) and decrease exponentially during a night of sleep (solid lines). This study analyzed data from NREM1, when SWA, a key electrophysiological marker of synaptic strength, is highest. The study hypothesis was that synaptic homeostasis, as indexed by NREM1 SWA, would differ between groups at baseline (left), would be modulated by ketamine (right), and would be more pronounced in TRD patients who responded to ketamine (orange dashed line, right) versus non-responders (purple dashed line, right). Representative hypnograms illustrating sleep architecture (stages) across the night are also depicted for reference. HV healthy volunteer, NREM1 the first NREM sleep period, PSG polysomnography, SWA slow-wave activity, TRD treatment-resistant depression.

Infusions began at approximately 10:30 AM, and post-treatment sleep was recorded that night, starting around 11 PM to match habitual sleep patterns; lights-on was set at 7 AM. Participants were instructed to avoid naps, especially during the three days before and after PSG nights, as naps reduce sleep pressure buildup and may decrease SWA in subsequent nighttime sleep. To maintain the non-napping protocol, participants were restricted from bed use during daytime hours and encouraged to engage in board games, puzzles, and art activities. Supervision by nursing staff and research fellows also helped participants adhere to non-napping requirements. Analyses focused on normalized slow-wave activity during the first NREM episode (NREM1 SWA), along with standard sleep architecture measures (TST, SE, WASO, SWS, SL). Figure 1 outlines the study timeline, including lights-out/lights-on scheduling and the timing of MADRS ratings and infusions.

### Statistical analyses

A linear mixed-effects model analysis evaluated NREM1 SWA changes across conditions (i.e., baseline, ketamine, placebo) for TRD and HV participants who received ketamine (n = 91 TRD patients and n = 21 HVs) and placebo (n = 62 TRD patients and n = 19 HVs). Condition (baseline, ketamine, placebo) was included as a fixed effect, with a random intercept for subject. SWA values were z-scored prior to modeling to standardize the outcome across participants. To reduce the influence of extreme values, observations exceeding |z | >3 were excluded prior to model fitting (two outliers). Model assumptions were evaluated by inspecting residual distributions and Lilliefors tests of residual normality. Further details regarding the statistical analyses can be found in the Supplement.

A two-sided p-value of 0.05 was considered statistically significant after Bonferroni corrections were made to individual p-values to account for multiple comparisons throughout the analyses. All statistical analyses were performed using GraphPad Prism version 10.5.0 for Windows, GraphPad Software, Boston, Massachusetts (www.graphpad.com), MATLAB R2024a (Mathworks, Natick, MA), and RStudio (version 2025.09.2 + 418; Posit Software, Boston, MA).

## Results

### Baseline NREM1 SWA and PSG: TRD patients versus HVs

**Fig. 2:**
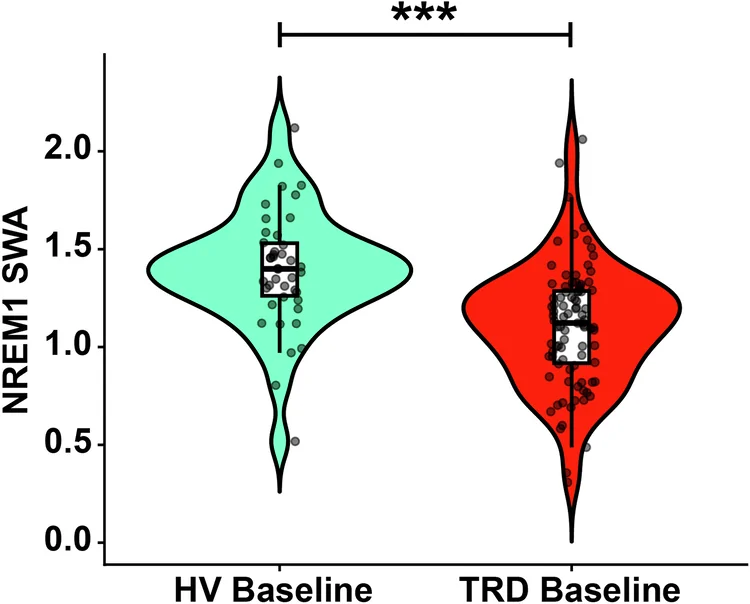
Slow-wave activity (SWA) during the first period of non-rapid eye movement sleep (NREM1) in patients with treatment-resistant depression (TRD, red) versus healthy volunteers (HVs, cyan) at baseline. Violin plots display the distribution of SWA values at baseline. Individual data points are overlaid, and the embedded boxplots indicate the median and interquartile range. Baseline SWA was significantly lower in TRD patients compared to HVs (***p < 0.0001).

Compared to HVs, TRD patients exhibited significantly lower NREM1 SWA at baseline (HVs: 1.40 ± 0.05; TRD: 1.11 ± 0.03, mean ± SEM; Welch’s *t*test, t = −5.17, p < 0.0001) (Fig. 2). At baseline, TRD patients exhibited significantly reduced TST (379.98 ± 7.95 vs 411.25 ± 7.06 min; Holm-Bonferroni p = 0.004) and SE (84.37 ± 1.54% vs 90.17 ± 1.48%; Holm-Bonferroni p = 0.02), along with significantly prolonged SL (30.19 ± 4.93 vs 13.94 ± 3.11 min; Holm-Bonferroni p = 0.02). No other sleep variables showed significant changes (Table S2).

### NREM1 SWA and PSG comparisons across conditions

**Fig. 3:**
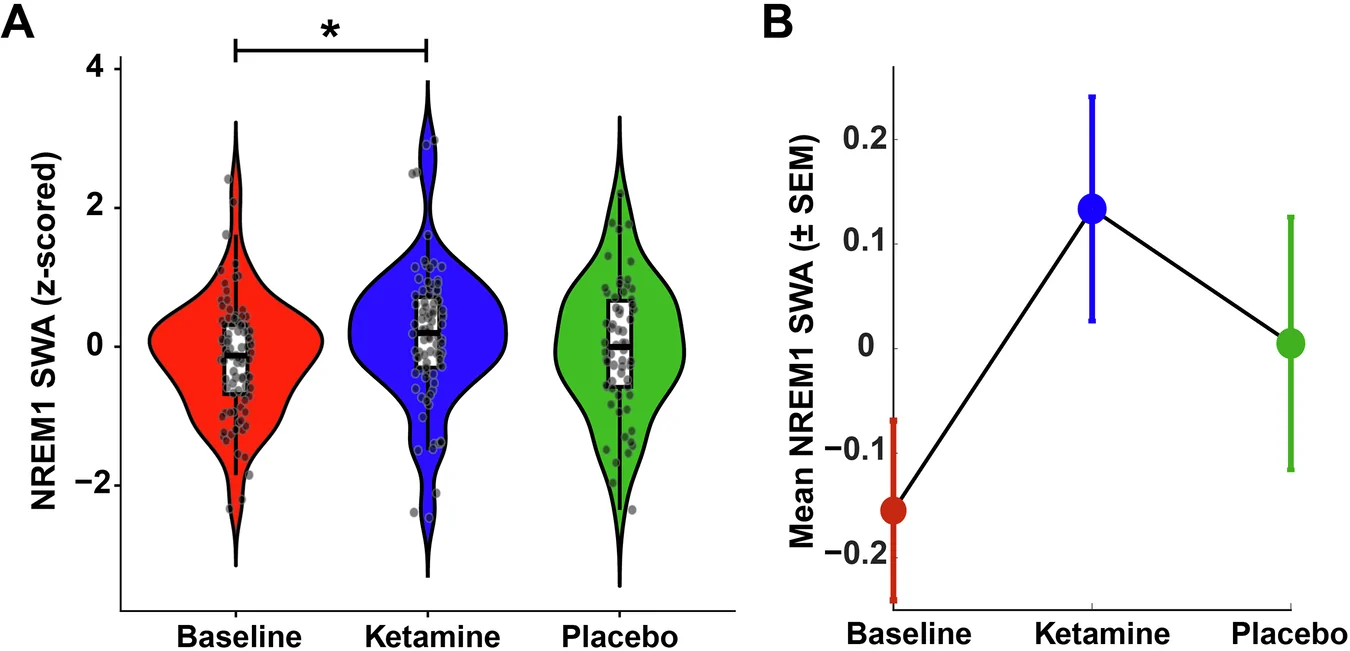
Slow-wave activity (SWA) during the first period of non-rapid eye movement sleep (NREM1) across conditions in treatment-resistant depression (TRD) patients. **A** Violin plots illustrate the distribution of z-scored SWA values at baseline (red), post-ketamine administration (blue), and post-placebo administration (green). Individual data points are overlaid, and the embedded boxplots indicate the median and interquartile range. **B** Mean ± SEM of z-scored NREM1 SWA across conditions. SWA significantly increased following ketamine compared to baseline (β = 0.292, p = 0.004, Bonferroni-corrected p = 0.012). *p = 0.01.

A linear mixed-effects model was implemented to assess changes in NREM1 SWA across baseline, ketamine, and placebo conditions. In TRD patients, effect of condition was significant (F(2,239) = 4.29, p = 0.015), and NREM1 SWA significantly increased post-ketamine compared to baseline (β = 0.292, SE = 0.102, 95%CI [0.089,0.494], p = 0.004, Bonferroni-corrected p = 0.012). In contrast, placebo did not significantly differ from baseline (β = 0.0698, SE = 0.117, t = 0.594, p = 0.553, Bonferroni-corrected p = 1.00), and ketamine did not differ from placebo (β = 0.222, SE = 0.118, t = 1.880, p = 0.061, Bonferroni-corrected p = 0.184) (Fig. 3). Post-ketamine, TRD participants showed significant increases in TST (379.98 ± 7.95 to 401.67 ± 5.65 min; Holm-Bonferroni p = 0.001) and SE (84.37 ± 1.54% to 87.57 ± 1.11%; Holm-Bonferroni p = 0.048), along with a significant reduction in SL (30.19 ± 4.93 to 19.93 ± 2.41 min; Holm-Bonferroni p = 0.048). No other sleep variables showed significant changes (Table S3).

As a secondary analysis, a linear mixed-effects model revealed no effect of condition on NREM1 SWA in HVs (F(2,79) = 0.11, p = 0.898). Neither ketamine (β = 0.015, p = 0.938) nor placebo (β = −0.080, p = 0.698) significantly differed from baseline. Consistent with this, no significant changes in other sleep variables were observed following ketamine relative to baseline in HVs (Table S4).

For TRD patients classified as ketamine responders, NREM1 SWA increased significantly post-treatment. Mean z-scored NREM1 SWA rose from −0.279 ± 0.127 (SEM) at baseline to 0.101 ± 0.151 post-ketamine (paired *t*test, t(36) = 2.617, p = 0.013, Bonferroni-corrected p = 0.026), and non-responders showed a non-significant trend between baseline ( − 0.099 ± 0.117) and post-ketamine (0.223 ± 0.173; paired *t*test, t(52) = 1.963, p = 0.0550, Bonferroni-corrected p = 0.110) (Fig. S2).

An exploratory analysis examined whether the ketamine response was associated with changes in NREM1 SWA. Baseline MADRS scores did not differ between future responders (32.51 ± 0.74) and non-responders (33.79 ± 0.61; p = 0.40), but post-treatment scores decreased to 9.54 ± 0.68 and 28.55 ± 0.94, respectively, with both groups showing significant improvement from baseline (both p < 0.0001) (Table S5). In an additional exploratory analysis of ECT-resistant patients (n = 17), a paired *t*-test showed no significant change in SWA post-ketamine (t(16) = 1.76, p = 0.097, 95%CI [ − 0.040,0.439]).

In sensitivity analyses adjusting for BMI and severity of baseline insomnia in TRD participants, neither BMI (β = 0.001, SE = 0.015, 95%CI [ − 0.029,0.031], p = 0.947, partial η²<0.001) nor severity of baseline insomnia (β = 0.043, SE = 0.055, 95%CI [ − 0.065,0.152], p = 0.430, partial η² = 0.004) was significantly associated with NREM1 SWA. Exploratory interaction models also showed no significant Condition×BMI (p = 0.640) or Condition×Insomnia interaction (p = 0.659). Similarly, in HVs, BMI was not significantly associated with NREM1 SWA (β = 0.0068, 95%CI [ − 0.061,0.074], p = 0.841). In the combined TRD/HV model adjusted for BMI, BMI remained non-significant (β = −0.0026, 95%CI [ − 0.024,0.019], p = 0.808), while the main effect of group remained significant (p < 0.001), indicating lower NREM1 SWA in TRD participants across conditions.

### Age moderation of ketamine-related NREM1 SWA changes (exploratory)

**Fig. 4:**
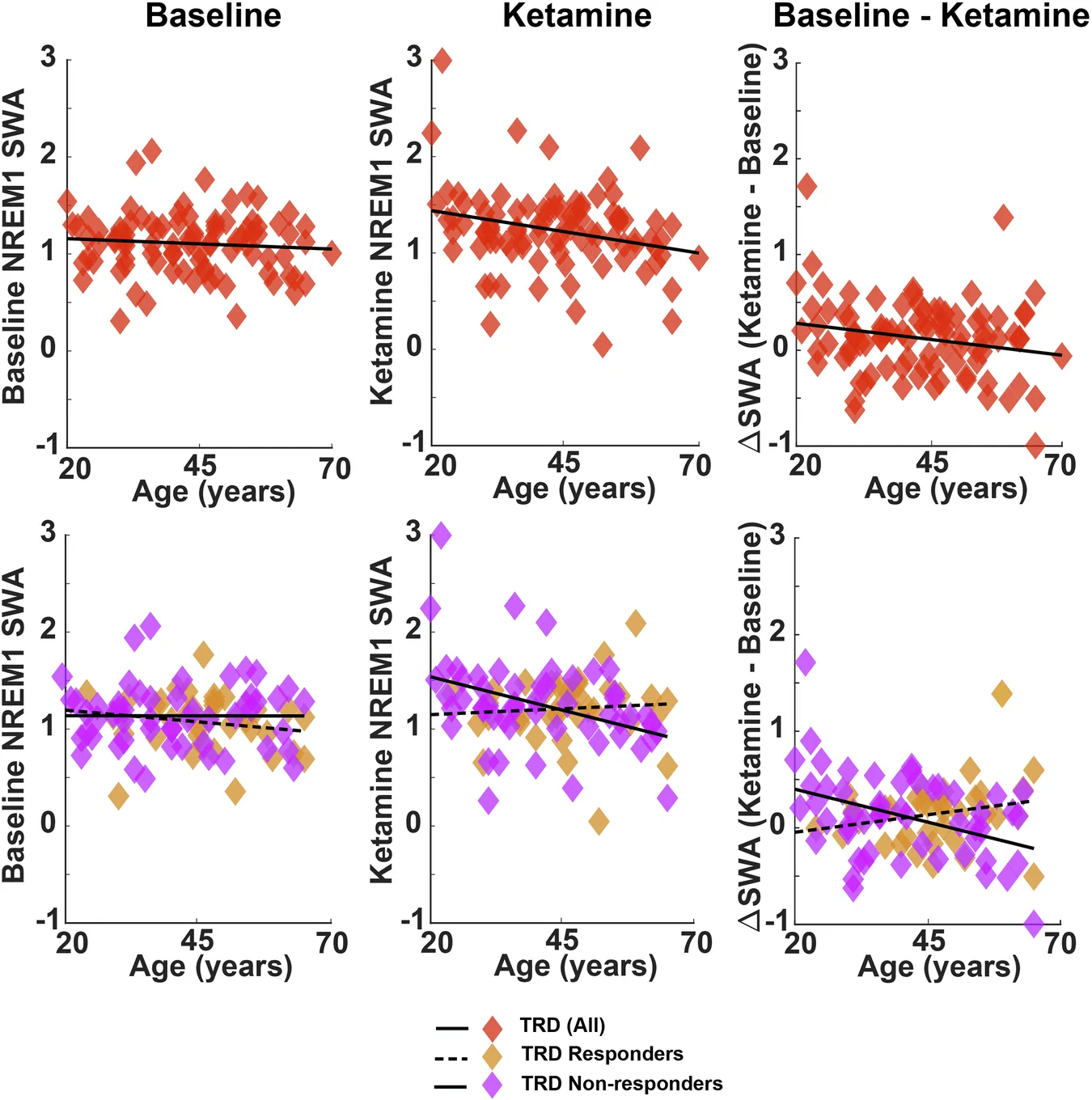
Age moderated ketamine-related changes in the first period of non-rapid eye movement sleep (NREM1) slow-wave activity (SWA) in treatment-resistant depression (TRD). Top row: Scatterplots show baseline NREM1 SWA, post-ketamine SWA, and ketamine-related change (ΔSWA = ketamine−baseline) plotted against age in TRD patients (red diamonds), with simple linear regression fits overlaid (red lines). Baseline SWA was not significantly associated with age (p = 0.409), whereas ketamine-session SWA decreased with increasing age (p = 0.012) and ΔSWA showed a modest age-related decline (p = 0.0467), indicating reduced ketamine-induced enhancement of early-night SWA in older TRD patients. Bottom row: The same analyses stratified by clinical response status show responders (orange) and non-responders (purple). While baseline SWA was not significantly associated with age in either subgroup, age was significantly related to post-ketamine SWA (p = 0.0053) and ΔSWA (p = 0.0032) in non-responders but not responders, with significant responder-group differences in slopes for post-ketamine SWA (p = 0.0417) and ΔSWA (p = 0.0054).

Age-related effects on NREM1 SWA were examined in TRD participants at baseline, following ketamine, and for ketamine-related change (ΔNREM1 SWA=ketamine−baseline). Baseline NREM1 SWA was not significantly associated with age (β = −0.002, p = 0.409; R² = 0.008); however, post-ketamine NREM1 SWA decreased significantly with increasing age (β = −0.009, p = 0.012; R² = 0.069), and ΔNREM1 SWA also showed a modest age-related decline (β = −0.007, p = 0.047; R² = 0.044), indicating reduced ketamine-related enhancement of early-night SWA in older TRD patients (Fig. 4, top). Consistent with these findings, a linear mixed-effects model found a significant main effect of ketamine (β = 0.415, SE = 0.147, 95%CI [0.126,0.705], p = 0.005, partial η²=0.043) as well as a significant Condition×Age interaction (β = −0.007, SE = 0.0033, 95%CI [ − 0.0131, − 0.0002], p = 0.043, partial η²=0.023), confirming that the ketamine-related increase in NREM1 SWA diminished with increasing age. The main effect of age at baseline was not significant (β = −0.0021, SE = 0.0030, 95%CI [ − 0.0081,0.0038], p = 0.477, partial η²=0.003). The age-moderation effect remained significant after adjusting for sex; specifically, the Condition×Age interaction remained significant (β = −0.0067, SE = 0.0033, 95%CI [ − 0.0131, − 0.0002], p = 0.043, partial η²=0.023), and the main effect of ketamine also remained significant (β = 0.415, SE = 0.147, 95%CI [0.126,0.705], p = 0.005, partial η²=0.043). Sex itself was not significantly associated with NREM1 SWA in this model (β = −0.079, 95%CI [ − 0.205,0.047], p = 0.218, partial η²=0.009).

As a secondary evaluation, age-related effects on NREM1 SWA were examined in HVs and compared with TRD patients. In HVs, a linear mixed-effects model showed no significant effect of ketamine on NREM1 SWA (β = −0.084, SE = 0.182, 95%CI [ − 0.447,0.279], p = 0.646, partial η²=0.004), no significant main effect of age (β = −0.0048, SE = 0.0039, 95%CI [ − 0.0127,0.0031], p = 0.228, partial η²=0.025), and no evidence of age moderation of ketamine-related NREM1 SWA change (Condition×Age interaction: β = 0.0023, SE = 0.0051, 95%CI [ − 0.0078,0.0125], p = 0.647, partial η²=0.004). These findings remained unchanged after adjusting for sex; ketamine was not associated with NREM1 SWA (β = −0.077, SE = 0.183, 95%CI [ − 0.443,0.289], p = 0.676, partial η²=0.003), age was not significantly associated with NREM1 SWA (β = −0.0048, SE = 0.0039, 95%CI [ − 0.0126,0.0030], p = 0.223, partial η²=0.025), and the Condition×Age interaction remained non-significant (β = 0.0021, SE = 0.0051, 95%CI [ − 0.0082,0.0124], p = 0.682, partial η²=0.003). Sex was also not significantly associated with NREM1 SWA in HVs (β = −0.047, 95%CI [ − 0.204,0.110], p = 0.552, partial η²=0.006). Consistent with this, in combined regression models that included group (HV vs TRD) and an Age×Group interaction, no significant interactions were observed for baseline NREM1 SWA (β = 0.0026, p = 0.605), ketamine-session NREM1 SWA (β = −0.0100, p = 0.253), or ΔNREM1 SWA (β = −0.0060, p = 0.482). Thus, although age-related associations were observed in TRD, the Age×Group interaction tests indicated that the age–NREM1 SWA relationships did not significantly differ between TRD and HV participants (Fig. S3).

Exploratory analyses further examined whether antidepressant response status influenced age-related associations with NREM1 SWA. These responder vs non-responder comparisons were conducted post-hoc and were not included as interaction terms in the primary linear mixed-effects models. In TRD patients, responder status moderated the relationship between age and ketamine-session NREM1 SWA outcomes (Fig. 4, bottom). No significant association was observed between baseline NREM1 SWA and age in either responders (β = −0.005, p = 0.306, R² = 0.030) or non-responders (β = −0.0001, p = 0.988, R² < 0.001), and slopes did not differ between groups (β = −0.0048, SE = 0.0060, 95%CI [ − 0.0166,0.0071], p = 0.427, partial η²=0.007). After adjusting for sex, the Age×Response interaction for baseline NREM1 SWA remained non-significant (β = −0.0039, SE = 0.0061, 95%CI [ − 0.0160,0.0082], p = 0.523, partial η²=0.005), and sex was not significantly associated with baseline NREM1 SWA (p = 0.452). Post-ketamine, NREM1 SWA was not associated with age in responders (β = 0.002, p = 0.665, R² = 0.005) but showed a significant negative association with age in non-responders (β = −0.014, p = 0.005; R² = 0.143), with a significant slope difference between responders and non-responders (β = 0.0161, SE = 0.0078, 95%CI [0.0006,0.0317], p = 0.042, partial η²=0.047). This responder-status moderation remained significant after adjusting for sex (Age×Response: β = 0.0185, SE = 0.0079, 95%CI [0.0029,0.0342], p = 0.021, partial η²=0.061), while sex was not significantly associated with post-ketamine NREM1 SWA (p = 0.104). Similarly, ketamine-related change in NREM1 SWA (ΔSWA = ketamine−baseline) was not significantly associated with age in responders (β = 0.007, p = 0.175, R² = 0.052), whereas ΔNREM1 SWA decreased significantly with increasing age in non-responders (β = −0.014, p = 0.003; R² = 0.158), and slopes differed significantly between responder groups (β = 0.0209, SE = 0.0073, 95%CI [0.0063,0.0354], p = 0.005, partial η²=0.086). The Age×Response interaction for ΔNREM1 SWA also remained significant after adjusting for sex (β = 0.0224, SE = 0.0074, 95%CI [0.0077,0.0372], p = 0.0034, partial η²=0.097), indicating that age-related attenuation of ketamine-associated SWA change was strongest in non-responders. Sex was not significantly associated with ΔNREM1 SWA (p = 0.265).

## Discussion

This study is the first to examine NREM1 SWA as a neurobiological marker of sleep homeostasis in TRD using a relatively large sample size and a rigorous, placebo-controlled, crossover design. Importantly, it includes a comparison group of HVs, providing a more robust framework for interpreting group differences in NREM1 SWA and its modulation by ketamine. At baseline, TRD patients exhibited significantly less SWA during NREM1 sleep than HVs. These findings replicate prior work from our group demonstrating reduced early night delta power in TRD [13], while extending those observations by leveraging a larger baseline HV sample, incorporating a ketamine versus placebo comparison within the TRD group, and examining ketamine effects in HVs who received the drug. Collectively, these results support the hypothesis that TRD is characterized by impaired sleep homeostasis, contributing to NREM1 SWA deficits.

Prior work in MDD suggested that inadequate accumulation of sleep pressure during wakefulness reflected impaired synaptic function [16, 28] or reduced neuronal synchronization [29]. Consistent with this literature, the current study reports lower baseline NREM1 SWA in TRD patients compared with HVs. Importantly, sensitivity analyses indicated that this group-related SWA difference was not explained by BMI and, in TRD patients, neither BMI nor baseline insomnia severity significantly accounted for NREM1 SWA. Within the framework of the synaptic homeostasis hypothesis [5], reduced NREM1 SWA may reflect impaired synaptic downscaling and disrupted cortical network coordination relevant to depression [30]. Because slow waves depend on coordinated activity across large scale cortical and thalamocortical network synchronization, reduced SWA may index broader deficits in network synchronization [3, 4]. Following ketamine administration, NREM1 SWA increased in TRD patients, but only among treatment responders, suggesting that ketamine may normalize impaired sleep homeostasis and synaptic plasticity in individuals capable of clinical response, consistent with prior findings [14] linking ketamine to increased SWA and synaptic plasticity.

**Fig. 5:**
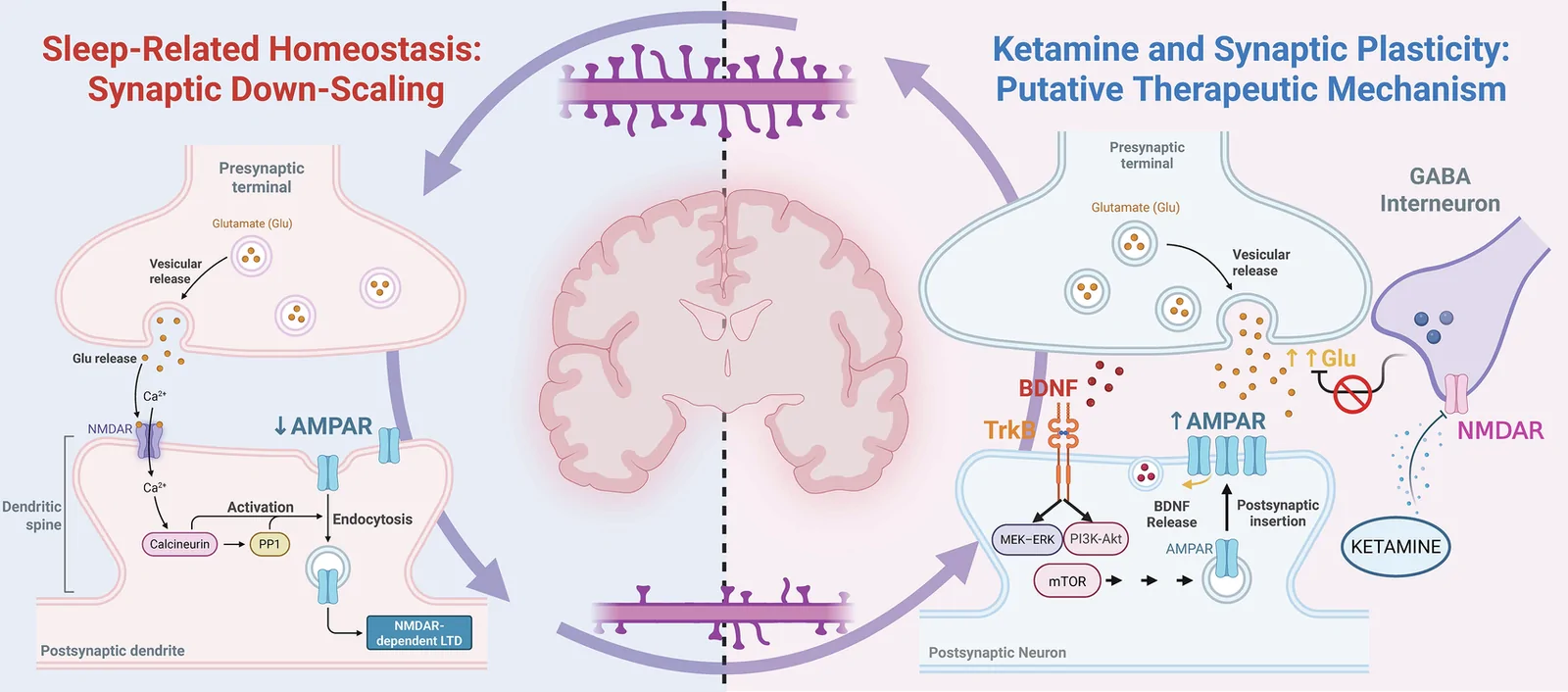
Contrasting models of synaptic plasticity: sleep-related homeostasis versus ketamine-induced synaptogenesis in depression. (Left) Schematic of sleep-dependent synaptic homeostasis. During NREM SWS, accumulated homeostatic pressure is hypothesized to drive global synaptic downscaling. This NMDAR-dependent LTD is mediated by calcium influx that activates phosphatases like calcineurin and PP1, leading to the dephosphorylation and endocytosis of AMPARs and a reduction in overall synaptic strength. (Right) Two leading hypotheses for ketamine’s rapid antidepressant effects. 1) Disinhibition hypothesis: Ketamine antagonizes NMDARs, particularly on GABA-ergic interneurons, causing a disinhibitory surge in presynaptic glutamate release. 2) Direct inhibition hypothesis: ketamine blocks tonic NMDAR activation by circulating glutamate, reducing suppression of eEF2 and boosting synaptic protein synthesis. Both theories converge on increased AMPAR transmission, which activates BDNF. This glutamate burst activates postsynaptic AMPARs, triggering the release of BDNF. Subsequent activation of the TrkB receptor and downstream signaling cascades, including MEK-ERK, PI3K-Akt, and mTOR, promotes the synthesis of synaptic proteins and the insertion of new AMPARs, driving rapid synaptogenesis. Akt protein kinase B, AMPAR α-amino-3-hydroxy-5-methyl-4-isoxazolepropionic acid receptor, BDNF brain-derived neurotrophic factor, eEF2 eukaryotic elongation factor 2, ERK extracellular signal-regulated kinase, GABA gamma-aminobutyric acid, Glu glutamate, LTD long-term depression, MEK mitogen-activated protein kinase kinase, mTOR mechanistic target of rapamycin, NMDAR N-methyl-D-aspartate receptor, NREM non-rapid eye movement, PI3K phosphoinositide 3-kinase, PP1 protein phosphatase 1, SWS slow wave sleep, TrkB tropomyosin receptor kinase B.

Ketamine exerts rapid antidepressant effects in 30-50% of TRD patients, though the underlying mechanisms remain incompletely understood [31]. Current models emphasize direct NMDA receptor inhibition and or preferential disinhibition of pyramidal neurons via gamma aminobutyric acid (GABA)-ergic interneurons—converging on enhanced α-amino-3-hydroxy-5-methyl-4-isoxazolepropionic acid receptor (AMPAR) signaling and downstream activation of BDNF–mechanistic target of rapamycin complex 1 (mTORC1) pathways essential for synaptic plasticity [32–34] (Fig. 5). Additional mechanisms, including modulation of opioid and insulin signaling and suppression of lateral habenula bursting, likely also contribute [35–37]. Regardless of the precise molecular targets, our findings suggest that improved sleep physiology, particularly enhanced NREM1 SWA, may underlie ketamine’s therapeutic effects. The selective increase in NREM1 SWA among responders raises the possibility that sleep-related plasticity is either a downstream marker or a mechanistic contributor to antidepressant response, consistent with evidence that BDNF signaling can directly modulate early SWA during subsequent sleep [38]. These mechanisms are consistent with broader models of antidepressant action that propose a two-stage process: rapid modulation of network excitability followed by slower network rewiring [39]. Under this framework, the increase in NREM1 SWA observed among ketamine responders (Fig. S2) might reflect an early network effect that supports synaptic renormalization and neuronal synchronization, thereby enabling longer-term circuit reorganization and sustained antidepressant response.

Sleep plays a central role in regulating synaptic strength and plasticity [5, 7]. Although sleep deprivation can transiently increase cortical excitability and even alleviate depressive symptoms in some patients [40], it ultimately disrupts synaptic plasticity and memory, underscoring the importance of sleep for synaptic recalibration [7]. In this context, the absence of increased NREM1 SWA in ketamine non-responders may reflect impaired synaptic renormalization due to insufficient wake-related potentiation or reduced capacity for large-scale network synchrony. Because SWA indexes the brain’s need for plastic recovery, low baseline NREM1 SWA in non-responders may indicate a limited plasticity reserve that constrains ketamine-induced network reorganization [4]. In contrast, ketamine responders in this study exhibited increased NREM1 SWA, consistent with restored synaptic synchrony and enhanced plasticity.

Beyond enhancing NREM1 SWA, ketamine also appeared to improve overall sleep architecture. At baseline, TRD patients showed reduced TST and SE as well as prolonged SL compared with HVs. Post-ketamine, TST and SE increased, and SL decreased, in TRD patients, supporting its treatment efficacy for sleep. These sleep effects may reflect ketamine’s ability to engage glutamatergic and homeostatic mechanisms that promote sleep depth and efficiency, consistent with reports linking ketamine-induced enhancements in SWA and macro-architectural sleep measures to mood improvement [41]. Prior work from our laboratory demonstrated that ketamine increased both REM sleep duration and REM density in TRD patients, with REM density in the first REM period predicting antidepressant response [42]. In a separate study, ketamine was also associated with concurrent increases in REM sleep and early night SWA [14]. These effects contrast with those of conventional antidepressants such as clomipramine, which primarily increase SWA by suppressing REM [43]. Together, these findings suggest that ketamine’s antidepressant effects may stem from coordinated enhancement of both REM and NREM processes, recalibrating dysregulated cortical circuits and restoring sleep-dependent synaptic homeostasis.

Overall, ketamine’s antidepressant effects likely result from multiple converging mechanisms—including NMDA receptor antagonism, BDNF/AMPAR/mTORC1 signaling, and sleep-related plasticity restoration—which are reflected in enhanced NREM1 SWA among treatment responders. While our study cannot resolve causality, the observed SWA changes support the idea that sleep may play a key role in therapeutic response to ketamine.

In HVs, ketamine did not alter NREM1 SWA or other parameters, suggesting a ceiling effect—that is, synaptic potentiation during wakefulness may already be saturated. Kuhn and colleagues [7] found that sleep recalibrates synaptic strength to maintain neural efficiency; in HVs, this process may already operate optimally, leaving little room for further SWA increases. This interpretation is consistent with our observation that HVs showed no significant differences in NREM1 SWA between post-ketamine and post-placebo nights, suggesting that ketamine’s capacity to enhance SWA may depend on the presence of impaired sleep homeostasis, as observed in TRD. However, these findings must be interpreted with caution due to the relatively small HV sample size. Future studies with larger healthy cohorts are required to confirm whether this lack of SWA modulation is a robust characteristic of the non-depressed brain. In contrast, in individuals with intact sleep regulation, ketamine produced no measurable changes in NREM1 SWA relative to placebo.

Our study also found that age moderated ketamine-related enhancement of NREM1 SWA in TRD patients, such that ketamine-induced increases in NREM1 SWA diminished with increasing age, particularly among non-responders (Fig. 4). This pattern is consistent with evidence that SWA declines with aging and reflects reduced synaptic plasticity and cortical homeostasis in older adults [13, 19]. This effect remained significant after adjusting for sex, suggesting that the observed age-related attenuation was not driven by sex differences in NREM1 SWA. Given that ketamine’s antidepressant effects are thought to involve synaptic potentiation and sleep-dependent plastic processes, attenuated SWA enhancement with age may represent a neurophysiological mechanism contributing to reduced treatment responsiveness in older TRD patients. However, because responder-stratified age analyses were exploratory and subgroup sizes were limited, these findings should be interpreted cautiously and replicated in larger samples.

Collectively, the results suggest that ketamine selectively normalized disrupted sleep homeostasis in depression rather than uniformly enhancing sleep. Despite these promising results, several limitations warrant attention. First, despite the overall large size of the sample, the number of ketamine responders was smaller than the number of non-responders, which may limit response-specific conclusions. Second, the inclusion of both MDD and bipolar depression within the TRD sample introduces diagnostic heterogeneity, which was not examined as a moderating factor. Third, the effect of repeated ketamine dosing remains unknown. Finally, only a subset of HVs received ketamine, underscoring the need for replication in larger control samples.

## Conclusion

Taken together, the findings indicate that ketamine enhanced NREM1 SWA selectively in treatment-responsive TRD patients, supporting a sleep-based mechanism underlying its rapid antidepressant effects. The increase in NREM1 SWA observed in responders—absent in HVs—suggests partial normalization of impaired sleep homeostasis and/or restoration of neuronal connectivity necessary for effective synaptic homeostasis. Beyond SWA modulation, ketamine improved sleep continuity in TRD, reflected by increased TST and SE and reduced SL. The attenuation of ketamine-related NREM1 SWA enhancement with advanced age further suggests that sleep-dependent plasticity mechanisms may diminish over the lifespan. Collectively, these findings underscore a mechanistic role for sleep regulation in both the pathophysiology and treatment of TRD.

## Supplementary information

Supplement

## Data Availability

De-identified participant data and study materials will be shared within the Open Science Framework (osf.io) (DOI will be provided at the time of manuscript acceptance).
