# Supplementary material for: Modulation of early non-rapid eye movement slow wave activity by ketamine in treatment-resistant depression
Source: Neuropsychopharmacology. 2026 Jun 16;51(11):2008–15. doi: 10.1038/s41386-026-02465-4 (PMC13597491; doi:10.1038/s41386-026-02465-4)
Supplement: Supplementary file 1 [file 41386_2026_2465_MOESM1_ESM.docx]

*Hejazi et al — Modulation of Early Non-Rapid Eye Movement Slow Wave Activity by*

*Ketamine in Treatment-Resistant Depression*

**SUPPLEMENT**

**Supplementary Methods**

*Statistical Analysis*

Body mass index (BMI) and severity of insomnia symptoms assessed using the Hamilton Depression Rating Scale (HAM-D) insomnia items (available for 68 participants with treatment-resistant depression (TRD)) were also included as continuous covariates in sensitivity analyses of slow wave activity (SWA) during the first period of non-rapid eye movement (NREM1). To evaluate whether age moderated ketamine-related changes in NREM1 SWA, linear mixed-effects models were fitted separately within TRD and healthy volunteer (HV) groups. Models included fixed effects of condition (baseline and ketamine), age, and their interaction (Condition×Age), with a random intercept for subject to account for repeated measures (NREM1 SWA ~ Condition × Age + (1|Subject)). To address potential sex effects, sex was added as an additional fixed-effect covariate in sensitivity models examining age moderation of ketamine-related NREM1 SWA changes. Effect sizes for model terms were reported as partial eta-squared (partial η²), and 95% confidence intervals (CIs) were reported for fixed-effect estimates. Ketamine-related change in SWA (ΔNREM1 SWA=ketamine−baseline) was analyzed separately using linear regression models including age as a predictor. In addition, a combined regression model was fitted including group (TRD vs HV) and an Age×Group interaction term (NREM1 SWA~Age×Group) to test whether age-related changes in NREM1 SWA differed between groups. Exploratory responder versus non-responder analyses were conducted post hoc; responder status was examined in stratified models and, where appropriate, in interaction models testing Age×Responder effects. Post-hoc contrasts were used to compare ketamine vs baseline, ketamine vs placebo, and placebo vs baseline, with two-sided significance testing. Normality was assessed using the Lilliefors test; paired *t*-tests were applied for normally distributed differences, and Wilcoxon signed-rank tests were used otherwise. The sleep parameters analyzed included total sleep time (TST), sleep efficiency (SE), wake after sleep onset (WASO), slow wave sleep (SWS), and sleep latency (SL).

*Additional Nighttime Sleep Measures & EEG spectral analysis*

The following sleep-related variables of interest were also assessed via polysomnography (PSG): 1) sleep onset, defined as the first occurrence of stage 2 sleep; 2) TST, defined as total minutes of sleep from sleep onset to final morning wake-up; 3) SE, expressed as a percentage and calculated by dividing TST by time in bed (TIB) multiplied by 100 (SE=(TST/TIB)×100; 4) TIB, defined as the total duration spent in bed during the sleep study; 5) WASO, defined as total minutes awake after initial sleep onset; 6) SWS, defined as total minutes spent in stages 3 and 4 sleep; and 7) SL, defined as the total minutes between lights-out and sleep onset. Non-rapid eye movement (NREM) and REM sleep episodes were defined as previously described [1,2]. The minimum duration for NREM episodes was 15 minutes.

Electroencephalography (EEG) recordings were obtained from the following sites: F3-A2, F4-A1, C3-A2, C4-A1, O1-A1, and O2-A1. Additional channels included bilateral electrooculograms, and a submental electromyogram was used for sleep staging purposes. Signals were acquired using a Nihon Kohden Neurofax system (Sleep v. 05-50) at a 200 Hz sampling rate and analyzed using Polysmith software (v4.0 and v10.0; Nihon Kohden, Japan). Two independent raters, blinded to condition, scored 30-second epochs according to standard criteria [3].

Sleep EEG power spectral density analysis was performed for NREM. EEG data for each staged 30-second epoch from channels C3-A2 and C4-A1 were further divided into five-second epochs that were used as inputs to the Matlab function *pwelch* with default parameters (Hamming window, 50% overlap) and a Fast Fourier Transform (fft) equal to the number of total samples in the five-second epoch (1000 samples); this resulted in 0.2 Hz bin resolution power spectral density resolution. SWA (0.6-4 Hz) and high-frequency spectral bands (20-40 Hz) were created by averaging across the frequency bins of interest for each five-second epoch. Thirty-second NREM epochs were then recreated by averaging across the original epochs and across the C3 and C4 channels, but were automatically rejected if any of the five-second epochs were above five times the average of the SWA band or eight times the high frequency band for that channel across all NREM epochs for each participant. NREM analyses focused on normalized SWA for NREM1 (SWA NREM1), which was calculated by dividing SWA in the first NREM episode by total SWA across all of NREM; the goal was to assess ketamine’s neurophysiological impact on the characteristic sleep waveform in early sleep. Central derivations (C3-A2 and C4-A1) were used because they were consistently available across participants, whereas frontal leads were not. This approach is consistent with prior studies quantifying SWA or delta power from central electrodes [4-6] and captures global sleep-related SWA dynamics [3,7]. However, because SWA shows regional variation and is often frontally predominant [8,9], the present findings should be interpreted as reflecting central derivation-based SWA rather than full scalp topography.

**Supplementary References**

1	Feinberg I, Floyd TC. Systematic trends across the night in human sleep cycles. Psychophysiology. 1979;16(3):283-91.

2	Feinberg I, Maloney T, March JD. Precise conservation of NREM period 1 (NREMP1) delta across naps and nocturnal sleep: implications for REM latency and NREM/REM alternation. Sleep. 1992;15(5):400-3.

3	Rechtschaffen A, Kales A. A Manual of Standardized Terminology, Techniques and Scoring System for Sleep Stages of Human Subjects. (Brain Information Service/Brain Research Institute, University of California, Los Angeles, Calif., 1968).

4	Baker FC, Kahan TL, Trinder J, Colrain IM. Sleep quality and the sleep electroencephalogram in women with severe premenstrual syndrome. Sleep. 2007;30:1283-91.

5	Dijk DJ, Brunner DP, Beersma DG, Borbély AA. Electroencephalogram power density and slow wave sleep as a function of prior waking and circadian phase. Sleep. 1990;13:430-40.

6	Duncan WC, Sarasso S, Ferrarelli F, Selter J, Riedner BA, Hejazi NS, et al. Concomitant BDNF and sleep slow wave changes indicate ketamine-induced plasticity in major depressive disorder. Int J Neuropsychopharmacol. 2013;16(2):301-11.

7	Finelli LA, Borbély AA, Achermann P. Functional topography of the human nonREM sleep electroencephalogram. Eur J Neurosci. 2001;13:2282-90.

8	Cajochen C, Foy R, Dijk DJ. Frontal predominance of a relative increase in sleep delta and theta EEG activity after sleep loss in humans. Sleep Res Online. 1999;2:65-69.

9	Plante DT, Landsnes EC, Peterson MJ, Goldstein MR, Riedner BA, Wanger T, et al. Sex-related differences in sleep slow wave activity in major depressive disorder: a high-density EEG investigation. BMC Psychiatry. 2012;12:146.

**Supplementary Table S1.** Demographics

|  | **TRD (n = 91)** | **HV (n = 42)** | **p-value** |
| --- | --- | --- | --- |
| **Sex*** | 51 F, 40 M | 23 F, 19 M | – |
| **Age (years)** | 43.09 ± 12.61 (Mean ± SD) | 34.14 ± 10.75  (Mean ± SD) | **0.0001** |
| **MADRS Total Score at baseline** | 33.27 ± 4.49  (Mean ± SD) | 1.22 ± 1.38  (Mean ± SD) | **<0.0001** |

TRD: treatment-resistant depression; HV: healthy volunteer; F: female; M: male; MADRS: Montgomery-Åsberg Depression Rating Scale. Statistical significance after Bonferroni correction (p<0.05) is indicated in bold.

*self-reported

**Supplementary Table S2.** Baseline comparison of polysomnographic (PSG) variables between healthy volunteers (HVs; n=42) and patients with treatment-resistant depression (TRD; n=91)

| **PSG Variable** | **Baseline HV**  **(M ± SEM)** | **Baseline TRD**  **(M ± SEM)** | **Raw**  **p-value** | **Holm-Bonferroni adjusted**  **p-value** |
| --- | --- | --- | --- | --- |
| SWS (min) | 26.32 ± 3.53 | 22.70 ± 3.54 | 0.07 | 0.14 |
| WASO (min) | 28.70 ± 8.13 | 41.58 ± 4.55 | 0.09 | 0.09 |
| SE (%) | 90.17 ± 1.48 | 84.37 ± 1.54 | 0.005 | **0.02** |
| TST (min) | 411.25 ± 7.06 | 379.98 ± 7.95 | 0.0008 | **0.004** |
| SL (min) | 13.94 ± 3.11 | 30.19 ± 4.93 | 0.005 | **0.02** |

SWS: Slow Wave Sleep; WASO: Wake After Sleep Onset; SE: Sleep Efficiency; TST: Total Sleep Time; SL: Sleep Latency. Statistical significance after Holm-Bonferroni correction (p<0.05) is indicated in bold.

**Supplementary Table S3.** Comparison of polysomnographic (PSG) variables in treatment-resistant depression (TRD; n=91) at baseline versus post-ketamine.

| **PSG Variable** | **Baseline TRD**  **(M ± SEM)** | **Ketamine TRD**  **(M ± SEM)** | **Raw**  **p-value** | **Holm–Bonferroni adjusted**  **p-value** |
| --- | --- | --- | --- | --- |
| SWS (min) | 22.29 ± 3.56 | 24.91 ± 2.95 | 0.07 | 0.14 |
| WASO (min) | 41.60± 4.60 | 39.98 ± 4.90 | 0.49 | 0.49 |
| SE (%) | 84.37 ± 1.54 | 87.57 ± 1.11 | 0.01 | **0.04** |
| TST (min) | 379.98 ± 7.95 | 401.67 ± 5.65 | 0.0002 | **0.001** |
| SL (min) | 30.19 ± 4.93 | 19.93 ± 2.41 | 0.01 | **0.04** |

SWS: Slow Wave Sleep; WASO: Wake After Sleep Onset; SE: Sleep Efficiency; TST: Total Sleep Time; SL: Sleep Latency. Statistical significance after Holm-Bonferroni correction (p < 0.05) is indicated in bold.

**Supplementary Table S4.** Comparison of polysomnographic (PSG) variables in healthy volunteers (HVs; n=22) at baseline versus post-ketamine.

| **PSG Variable** | **Baseline**  **(Mean ± SEM)** | **Ketamine**  **(Mean ± SEM)** | **Raw**  **p-value** |
| --- | --- | --- | --- |
| SWS (min) | 31.07 ± 4.17 | 35.47 ± 4.22 | 0.35 |
| WASO (min) | 28.70 ± 8.13 | 30.69 ± 7.79 | 0.73 |
| SE (%) | 90.23 ± 2.21 | 89.45 ± 1.86 | 0.57 |
| TST (min) | 422.06 ± 9.73 | 408.14 ± 8.67 | 0.11 |
| SL (min) | 14.07 ± 4.68 | 14.88 ± 2.56 | 0.23 |

SWS: Slow Wave Sleep; WASO: Wake After Sleep Onset; SE: Sleep Efficiency; TST: Total Sleep Time; SL: Sleep Latency. No significant changes in sleep variables were observed following ketamine relative to baseline in HVs.

**Supplementary Table S5**. Montgomery-Asberg Depression Rating Scale (MADRS) score comparisons in patients with treatment-resistant depression (TRD) who were ketamine responders vs non-responders

| **Group** | **Baseline MADRS (Mean ± SEM)** | **Post-Ketamine MADRS (Mean ± SEM)** | **p-value** |
| --- | --- | --- | --- |
| Responders (n = 37) | 32.51 ± 0.74 | 9.54 ± 0.68 | **<0.0001** |
| Non-responders (n = 53) | 33.79 ± 0.61 | 28.55 ± 0.94 | **<0.0001** |

Statistical significance after Bonferroni correction (p < 0.05) is indicated in bold.

**Supplementary Figure S1. CONSORT Diagram for the slow-wave activity (SWA) Ketamine Study**

308 valid nights

133 baseline nights

175 post-infusion nights

64 nights from HVs (from one protocol: KMOA)

244 nights from patients with TRD (only from two specific protocols: KMOA* and BP^¥^)

64 nights (42 baseline and 22 ketamine) (KMOA=42)

244 nights [(91 baseline and 153 post-infusion, (ketamine: n=91, placebo: n=62)] from 91 TRD patients

(KMOA*= 64, BP^¥^ =27)

*Ketamine mechanism of action study (KMOA, NCT00088699)

^¥^ Bipolar study (BP, NCT01204918)

TRD: treatment-resistant depression: HV: healthy volunteers

**Supplementary Figure S2.** Comparison of slow-wave activity (SWA) during the first period of non-rapid eye movement sleep (NREM1) at baseline (BL) versus post-ketamine (K) in responders (left, orange) and non-responders (right, purple). Violin plots show the distribution of z-scored SWA values. Individual data points are overlaid, and the embedded boxplots indicate the median and interquartile range. **(Left)** In responders (n=37), SWA significantly increased following ketamine (two-tailed paired *t*-test: t(36)=2.617, p=0.013, Bonferroni-corrected p=0.0258). **(Right)** Non-responders (n=53) showed no significant change (two-tailed paired *t*-test: t(52)= 1.963, p=0.0550, Bonferroni-corrected p=0.110).

**Supplementary Figure S3**. Relationships between age and the first period of non-rapid eye movement sleep (NREM1) slow-wave activity (SWA) at baseline and post-ketamine, and ketamine-induced change in NREM1 SWA in treatment-resistant depression (TRD) and healthy volunteer (HV) participants. Scatterplots show individual SWA values plotted against age for HVs (cyan circles) and TRD patients (red diamonds), with separate simple linear regression fits overlaid (cyan=HV; red=TRD). Left: Baseline NREM1 SWA was not significantly associated with age in HVs (p=0.270) or TRD (p=0.409). Middle: Ketamine-session SWA showed a significant negative association with age in TRD (p=0.012) but not in HVs (p=0.795). Right: Ketamine-related change in SWA (ΔSWA=ketamine−baseline) decreased with age in TRD (p=0.047), whereas no significant association was observed in HVs (p=0.903). In combined regression models including an Age×Group interaction, no significant interactions were observed for baseline SWA, ketamine SWA, or ΔSWA, indicating that age-related associations did not significantly differ between TRD and HV participants.
